# Prediction model and scoring system for in-hospital mortality risk in infants with heart failure aged 1–36 months: A retrospective case-control study

**DOI:** 10.1016/j.heliyon.2025.e42110

**Published:** 2025-01-18

**Authors:** Meng Wei, Shuai Shang, Huasheng Lv, Xiaoyan Liang, Yanmei Lu, Baopeng Tang

**Affiliations:** a Department of Cardiac Function, The First Affiliated Hospital of Xinjiang Medical University, Urumqi, 830000, Xinjiang, China; b Xinjiang Key Laboratory of Cardiac Electrophysiology and Cardiac Remodeling, The First Affiliated Hospital of Xinjiang Medical University, Urumqi, 830000, Xinjiang, China

**Keywords:** Infants and young children, Heart failure, Death, Prediction model, Scoring system

## Abstract

**Background:**

Existing studies report very few factors influencing the mortality of infant and toddler heart failure patients during hospitalization. Due to its high mortality rate, it is an important health issue. Therefore, this study aims to explore the factors influencing infant and toddler heart failure patients during hospitalization, establish predictive models, and a mortality scoring table.

**Methods:**

The study ultimately included 544 cases of infant heart failure patients. They were randomly divided into a training set (380 cases) and a validation set (164 cases) in a ratio of 7:3. The training set was then further divided into the death group and the survival group for further analysis of indicators during hospitalization.

**Results:**

Using the Lasso regression method, this study selected the best 14 variables from 88 independent variables of infants and toddlers with heart failure. Multivariate Logistic regression results show that TP < 65 g/L (OR = 2.34), pH < 7.35 (OR = 2.79), and Respiratory rate (1–12 months: <30times/min and 13–36 months: <25times/min, OR = 2.34) are independent risk factors. The model evaluation results for the train and test sets of infant and toddler heart failure patients are as follows: C-index values for discrimination in the train and test sets are 0.721 and 0.728, respectively. Fit test calibration evaluations show P values of 0.9958 and 0.9998, both greater than 0.05, indicating good calibration. The AUC values for the train and test sets are 0.75 and 0.64, respectively, showing a good predictive effect of the model. The mortality scoring table divides patients in the train and test sets into low risk, moderate-risk, and high risk categories. Compared to the low risk group, the OR values for the occurrence of mortality in the Medium risk group and high risk group in the train set are 3.78 and 11.67, respectively; in the test set, the OR values for the moderate-risk group and high-risk group are 1.73 and 6.33, respectively.

**Conclusion:**

The predictive models and scoring tables established in this study have a good predictive role in assessing the risk of death in infant and toddler heart failure patients aged 1–36 months during hospitalization, providing clinical guidance and reference value.

## 1 Introduction

Heart failure is one of the common pediatric critical illnesses in clinical practice. Its definition is similar to that in adults, it is an abnormal change in heart structure and function caused by multiple reasons, leading to impaired ventricular contraction and/or relaxation function, insufficient cardiac output to meet the body's needs, and also causing disorders in neuroendocrine regulation, affecting the heart and various organs in the body, creating a complex clinical syndrome. Heart failure is one of the important causes of death in children [1]. A systematic review of heart failure in children and adolescents in the past 20 years reveals an incidence rate of 0.9/100000 to 7.4/100000 and a prevalence rate of approximately 83.3/100000, with various causes [2]. In the United States, the mortality rates of heart failure in children and adults are 5.8 % and 4.8 % respectively, with the mortality rate in children higher than that in adults, resulting in a heavy burden on both health and economics [3]. Heart failure can occur at any age in the fetus or childhood, with highly heterogeneous etiologies, which can be congenital or acquired. The causes vary across different age groups, with myocarditis, cardiomyopathy, severe arrhythmias, and metabolic diseases being potential causes of heart failure at any age. Infections, exercise, anemia, electrolyte disturbances, and acidosis are common factors that can trigger heart failure [4].

Just as described above, the causes of heart failure in infants are complex and have a high mortality rate, which inevitably causes us to pay high attention. For infants with acute right heart failure, the death rate during hospitalization can be as high as 5 %–17 % [5]. Therefore, in fact, the mortality rate of all infants with heart failure during hospitalization is higher than the above figure.

Due to the lack of a risk assessment tool for inpatients with heart failure among infants and young children, our hospital, a large regional medical center, serves patients aged 1–36 months from 15 different ethnic groups with comprehensive records and representation during hospitalization. Therefore, we can establish predictive models and scoring tables by utilizing existing patient information from various perspectives. By combining clinical characteristics, cardiac function indicators, and diagnostic results of pediatric patients, we can provide estimates of risks such as inpatient mortality rate to maximize the survival and quality of life of the patients. Similarly, by identifying high-risk patients, doctors can take more proactive monitoring and intervention measures, such as drug therapy, hemodynamic support, intensive care in the cardiac unit, to improve the survival rate of pediatric patients.

## 2 Methodology

### 2.1 Research design and study subjects

**Fig. 1:**
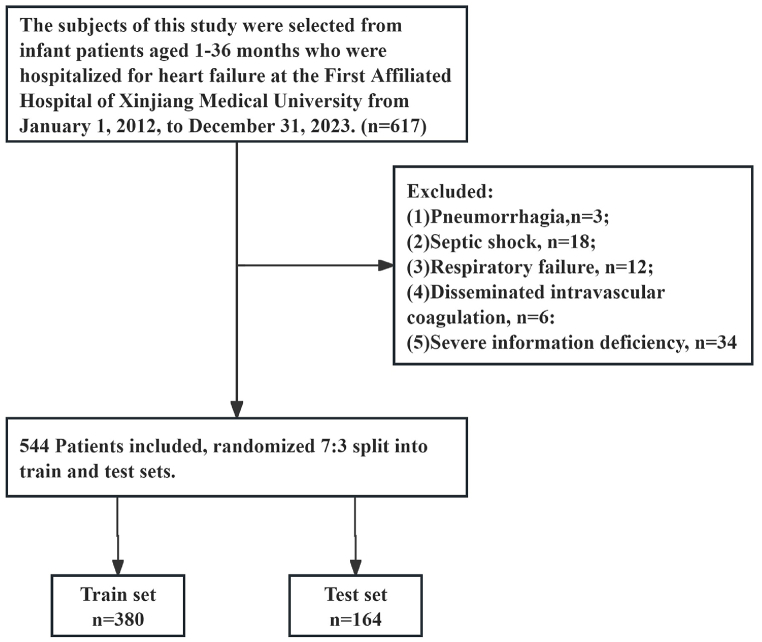
Flow chart of the screening of the study subjects.

This research design was approved by the Ethics Committee of the First Affiliated Hospital of Xinjiang Medical University, and this retrospective cohort study (ethical number: 231124-05) is a retrospective case-control study. The study subjects were infants and young children with heart failure admitted to the First Affiliated Hospital of Xinjiang Medical University from January 1, 2012 to December 31, 2023 (1–36 months), totaling 617 cases. Exclusion criteria were: pulmonary hemorrhage, septic shock, respiratory failure, disseminated intravascular coagulation, and patients with severe missing information. According to the above inclusion and exclusion criteria, patients were randomly divided into a train set (n = 380) and test set (n = 164) in a 7:3 ratio, detailed process shown in Fig. 1. As this study is retrospective in nature, informed consent for infants and their guardians was waived, which was also approved by the ethics committee.

### 2.2 Data collection

Clinical data were collected from the hospital records of infants with heart failure, including: (1) Demographic characteristics such as age, sex, ethnicity, number of hospitalizations, and New York Heart Function Class (NYHA); (2) Medical history, encompassing fever, shortness of breath or dyspnea, congenital heart disease, consciousness levels, oedema of the lower limbs, heart murmurs, moist rales, and repeated fever incidents; (3) Physical examination findings, including heart rate, temperature, respiratory rate, systolic and diastolic blood pressures (SBP and DBP, respectively), and Body Mass Index (BMI); (4) Medication history, covering beta blockers, angiotensin-converting enzyme inhibitors (ACEI), angiotensin receptor antagonists (ARB), angiotensin receptor-neprilysin inhibitors (ARNI), spironolactone, and furosemide; (5) Cardiac ultrasound examination results, detailing cardiac stroke volume (SV), cardiac output (CO), left atrial diameter (LAD), left ventricular end systolic and diastolic diameters (LVESD and LVEDD), left ventricular ejection fraction (LVEF), interventricular septal thickness, left ventricular posterior wall thickness, right atrial diameter (RAD), and right ventricular diameter. Blood laboratory tests, noting white and red blood cell counts (WBC and RBC), mean corpuscular volume (MCV), mean corpuscular hemoglobin concentration (MCHC), red cell distribution width coefficient of variation (RDW-CV), lymphocyte, monocyte, neutrophil, eosinophil, and basophil counts, hematocrit (Hct), hemoglobin, platelet count and volume (PLT and MPV), platelet distribution width (PDW), thrombocrit (PCT), liver function tests namely alanine and aspartate aminotransferases (ALT and AST), γ-glutamyltransferase (GGT), total and direct bilirubin (TBIL and DBIL), indirect bilirubin (IBIL), albumin (ALB), globulin (GLO), total protein (TP), albumin/globulin ratio (ALB/GLO), renal function markers such as creatinine, urea, uric acid (UA), lipid profile including total cholesterol (TC), triglycerides (TG), high and low density lipoprotein cholesterol (HDL-c and LDL-c), electrolytes such as potassium, sodium, chloride, calcium, phosphorus, magnesium, glucose, lactate dehydrogenase (LDH), alkaline phosphatase (ALP), cholinesterase (ChE), cystatin C (Cys-C), coagulation panels including thrombin time (TT), prothrombin time (PT), international normalized ratio (PT-INR), D-Dimer, pH, glycosylated serum protein (GSP), procalcitonin (PCT), N-terminal pro-brain natriuretic peptide (NT-ProBNP), creatine kinase (CK), and renal function tests like creatinine clearance rate (GCR) and glomerular filtration rate (GFR).

### 2.3 Study outcome events

The observation of this study is the in-hospital mortality outcome of patients with a primary diagnosis of heart failure who are infants and young children aged 1–36 months. All-cause mortality is defined as death caused by various reasons such as cancer, cardiovascular events, and respiratory infections during hospitalization.。

### 2.4 Statistical analysis

Using SPSS version 22.0 software (SPSS Inc., Chicago, IL, USA), R software (version 4.2.1; R Foundation for Statistical Computing, Vienna, Austria), the applied R packages include: glmnet [4.1.7], pROC [1.18.0], ggplot2 [3.3.6], rms [6.4.0], ResourceSelection [0.3–5], rmda [1.6], for data analysis. For continuous variables following a normal distribution, we used mean ± standard deviation for description, and for those meeting the conditions of *t*-test, we used independent-sample *t*-test; otherwise, we used the median and quartile range (25th, 75th percentile) representation [P50 (P25, P75)], and comparison between groups was done using the Wilcoxon Mann-Whitney test. For categorical variables, this study used n (%) for representation, and comparison of composition ratios within groups was done using the chi-square test or Fisher's exact probability method.

In this study, the patients collected 88 independent variables, using Lasso regression to add an L1 norm constraint term behind the cost function of the linear regression model. By controlling the parameter lambda, it can be used for variable selection and complexity adjustment. For high-dimensional data variable selection, feature selection, and subsequent construction of logistic regression models, ten-fold cross-validation was used. The selected result used the best evaluation index of lambda value (lambda.min) and selected the most appropriate variables. The specific diagnostic Lasso coefficient selection and variable selection trajectory are presented in the first part of the results.

After conducting Lasso regression screening, dummy variables were assigned to the preliminary variables. Subsequently, univariate logistic regression was applied to assess patient mortality during hospitalization. Variables with a P-value less than 0.1 were further subjected to multivariate logistic regression analysis. From these results, a logistic regression model was developed, facilitating the execution of a receiver operating characteristic (ROC) curve analysis. This analysis underscores the efficacy of combined indicators, utilizing the Area Under the Curve (AUC) to evaluate the predictive diagnostic performance.

Enhancing the Application of Decision Curve Analysis (DCA) for Evaluating the Predictive Efficacy of a Clinical Prediction Model. This study employs a Generalized Linear Model (GLM) to construct a binary logistic model, utilizes calibration analysis and visualization to depict the discrepancies between predicted probabilities and actual outcomes, and assesses the model's fit. A diagnostic nomogram is constructed by integrating predictive indices through scaled line segments, plotted on a plane in proportion to illustrate the interrelationships among variables within the predictive model. Model calibration is evaluated using the Hosmer-Lemeshow Goodness of Fit test.

Using multivariate regression results, this study established a risk score table for in-hospital mortality among infants with heart failure, based on The Framingham Study risk score functions [6]. Additionally, a score effect analysis was conducted. The significance level for all statistical methods employed in this study was set at α = 0.05.

## 3 Results

### 3.1 Using Lasso regression for preliminary data cleaning

**Fig. 2:**
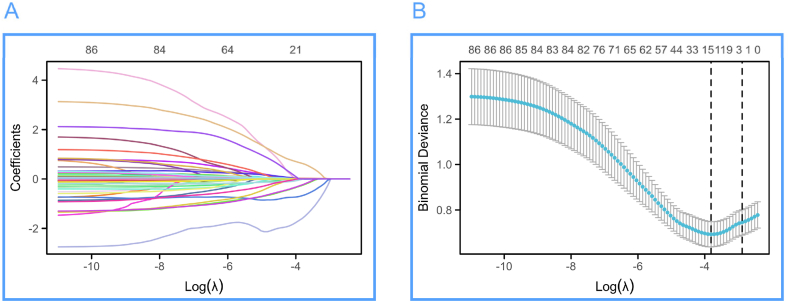
Variable trajectory and selection process diagram of Lasso regression. (A) Variable trajectory diagram of Lasso regression, (B) Selection flowchart of Lasso regression.

After Lasso regression, in this study, 14 variables were selected from the 88 independent variables of infant heart failure patients, including TP, WBC, Lymph, PCT, DBIL, K^+^, Mg^2+^, TT, pH, NT-ProBNP, Respiratory rate, Race, β-blocker, and ACEI/ARB/ARNI. The variable trajectory and selection process of Lasso regression are shown in Fig. 2.

### 3.2 Comparison of train and test sets for infants with heart failure

In this study, all data were split in a 7:3 ratio, randomly divided into a test set (164 cases) and a train set (380 cases). After comparison, there were no significant differences between the train set and the test set in indicators such as TP, WBC, Lymph, PCT, DBIL, K^+^, Mg^2+^, TT, pH, NT-ProBNP, Respiratory rate, Race, β-blocker, ACEI/ARB/ARNI, and death. There were 78 deaths during hospitalization in this study, with a survival rate of 466 cases, resulting in a high mortality rate of 14.34 % during hospitalization. The mortality rates in the train set and test set were 11.59 % and 15.53 %, respectively. After dummy variable assignment, there were still no differences between the above indicators in the train set and test set, demonstrating good allocation results. The P values in the above results were all >0.05. Details of the dummy variable assignments can be found in Table 1, and more information is available in Table 2, Table 3.

**Table 1:** Dummy variable assignment table for each variable.

Variables	Rules for assigning dummy variables
Death	No = 0, Yes = 1
Race	Other = 0, Han = 1
WBC, 10^9/L	[3.5, 9.5] = 0, <3.5 = 1, >9.5 = 2
Lymph, 10^9/L	[1.10, 3.20] = 0, <1.10= 1, >3.20 = 2;
PCT, ng/ml	[0, 0.5] = 0, >0.5 = 1
DBIL, umol/L	≤8.6 = 0, >8.6 = 1;
TP, g/L	[65, 85] = 0, <65 = 1, >85 = 2;
K^+^, mmol/L	[3.5, 5.3] = 0, <3.5 = 1, >5.3 = 2;
Mg^2+^, mmol/L	[0.75, 1.02] = 0, <0.75 = 1, >1.02 = 2;
TT, S	[16, 25] = 0, <16 = 1, >25 = 2;
pH	[7.35, 7.45] = 0, <7.35 = 1, >7.45 = 2;
NT-ProBNP, ng/L	[0, 125] = 0, (125, 1800) = 1, ≥1800 = 2;
Respiratory rate, times/min	1–12 months: [30, 40] = 0, <30 = 1, >40 = 2; 13–36 months: [25, 30] = 0, <25 = 1, >30 = 2;
β-blocker	No = 0, Yes = 1
ACEI/ARB/ARNI	No = 0, Yes = 1

**Table 2:** Comparison of original data between train and test sets of infant heart failure patients.

Variables	Total (n = 544)	test (n = 164)	train (n = 380)	t/Z/χ^2^	*P*
TP, Mean ± SD	60.20 ± 8.72	59.63 ± 8.72	60.44 ± 8.72	−0.99	0.323
WBC, M (Q₁, Q₃)	11.43 (7.93, 15.94)	11.59 (8.20, 15.94)	11.38 (7.85, 15.90)	−0.41	0.682
Lymph, M (Q₁, Q₃)	4.83 (3.00, 6.80)	4.92 (3.00, 6.90)	4.79 (3.00, 6.75)	−0.3	0.762
PCT, M (Q₁, Q₃)	0.31 (0.22, 0.41)	0.32 (0.22, 0.42)	0.31 (0.22, 0.41)	−0.65	0.515
DBIL, M (Q₁, Q₃)	1.73 (0.30, 3.55)	1.65 (0.30, 3.77)	1.81 (0.30, 3.48)	−0.09	0.932
K^+^, M (Q₁, Q₃)	4.19 (3.71, 4.56)	4.26 (3.69, 4.53)	4.18 (3.74, 4.59)	−0.17	0.868
Mg^2+^, M (Q₁, Q₃)	0.91 (0.84, 0.99)	0.91 (0.85, 1.00)	0.91 (0.84, 0.98)	−0.58	0.562
TT, M (Q₁, Q₃)	24.90 (22.30, 27.42)	25.20 (22.58, 27.22)	24.80 (22.20, 27.63)	−0.44	0.661
pH, M (Q₁, Q₃)	7.39 (7.31, 7.45)	7.40 (7.32, 7.45)	7.39 (7.31, 7.44)	−1.52	0.129
NT-ProBNP, M (Q₁, Q₃)	2842.50 (1547.75, 4568.25)	2878.50 (1496.25, 4264.00)	2822.00 (1585.00, 4576.25)	−0.34	0.734
Respiratory rate, M (Q₁, Q₃)	38.00 (30.00, 50.00)	40.00 (31.00, 52.00)	36.50 (30.00, 49.00)	−1.56	0.119
Death, n(%)				1.45	0.229
No	466 (85.66)	145 (88.41)	321 (84.47)		
Yes	78 (14.34)	19 (11.59)	59 (15.53)		
Race, n(%)				0.05	0.817
No	341 (62.68)	104 (63.41)	237 (62.37)		
Yes	203 (37.32)	60 (36.59)	143 (37.63)		
β-blocker, n(%)				0.23	0.631
No	387 (71.14)	119 (72.56)	268 (70.53)		
Yes	157 (28.86)	45 (27.44)	112 (29.47)		
ACEI/ARB/ARNI, n(%)				2.39	0.122
No	421 (77.39)	120 (73.17)	301 (79.21)		
Yes	123 (22.61)	44 (26.83)	79 (20.79)		

**Note.** t: *t*-test, Z: Mann-Whitney test, χ^2^: Chi-square test; SD: standard deviation, M: Median, Q₁: 1st Quartile, Q₃: 3rd Quartile.

**Table 3:** Comparison of dummy variable assignment between train and test sets of infant heart failure patients.

Variables	Total (n = 544)	Test (n = 164)	Train (n = 380)	χ^2^	*P*
Death, n(%)				1.45	0.229
No	466 (85.66)	145 (88.41)	321 (84.47)		
Yes	78 (14.34)	19 (11.59)	59 (15.53)		
Race, n(%)				0.05	0.817
Other	341 (62.68)	104 (63.41)	237 (62.37)		
Han	203 (37.32)	60 (36.59)	143 (37.63)		
WBC, n(%)				0.93	0.628
[3.5, 9.5]	182 (33.46)	50 (30.49)	132 (34.74)		
<3.5	16 (2.94)	5 (3.05)	11 (2.89)		
>9.5	346 (63.60)	109 (66.46)	237 (62.37)		
Lymph, n(%)				1.86	0.395
[1.10, 3.20]	133 (24.45)	43 (26.22)	90 (23.68)		
<1.10	18 (3.31)	3 (1.83)	15 (3.95)		
>3.20	393 (72.24)	118 (71.95)	275 (72.37)		
PCT, n(%)				0.16	0.689
[0, 0.5]	490 (90.07)	149 (90.85)	341 (89.74)		
>0.5	54 (9.93)	15 (9.15)	39 (10.26)		
DBIL, n(%)				0.22	0.639
≤8.6	502 (92.28)	150 (91.46)	352 (92.63)		
>8.6	42 (7.72)	14 (8.54)	28 (7.37)		
TP, n(%)				–	0.705
[65, 85]	141 (25.92)	38 (23.17)	103 (27.11)		
<65	399 (73.35)	125 (76.22)	274 (72.11)		
>85	4 (0.74)	1 (0.61)	3 (0.79)		
K^+^, n(%)				2.38	0.304
[3.5, 5.3]	420 (77.21)	132 (80.49)	288 (75.79)		
<3.5	84 (15.44)	24 (14.63)	60 (15.79)		
>5.3	40 (7.35)	8 (4.88)	32 (8.42)		
Mg^2+^, n(%)				1.24	0.538
[0.75, 1.02]	396 (72.79)	118 (71.95)	278 (73.16)		
<0.75	51 (9.38)	13 (7.93)	38 (10.00)		
>1.02	97 (17.83)	33 (20.12)	64 (16.84)		
TT, n(%)				–	0.407
[16, 25]	283 (52.02)	80 (48.78)	203 (53.42)		
<16	2 (0.37)	0 (0.00)	2 (0.53)		
>25	259 (47.61)	84 (51.22)	175 (46.05)		
pH, n(%)				2.73	0.255
[7.35, 7.45]	227 (41.73)	72 (43.90)	155 (40.79)		
<7.35	193 (35.48)	50 (30.49)	143 (37.63)		
>7.45	124 (22.79)	42 (25.61)	82 (21.58)		
NT-ProBNP, n(%)				2.71	0.258
[0, 125]	31 (5.70)	13 (7.93)	18 (4.74)		
(125, 1800)	10 (1.84)	4 (2.44)	6 (1.58)		
≥1800	503 (92.46)	147 (89.63)	356 (93.68)		
Respiratory rate, n(%)				1.68	0.431
1–12 months: [30, 40], 13–36 months: [25, 30]	210 (38.60)	59 (35.98)	151 (39.74)		
1–12 months: <30, 13–36 months: <25	68 (12.50)	18 (10.98)	50 (13.16)		
1–12 months: >40, 13–36 months: >30	266 (48.90)	87 (53.05)	179 (47.11)		
β-blocker, n(%)				0.23	0.631
No	387 (71.14)	119 (72.56)	268 (70.53)		
Yes	157 (28.86)	45 (27.44)	112 (29.47)		
ACEI, n(%)				2.39	0.122
No	421 (77.39)	120 (73.17)	301 (79.21)		
Yes	123 (22.61)	44 (26.83)	79 (20.79)		

**Note.** χ^2^: Chi-square test, -: Fisher exact.

### 3.3 Variable feature analysis of infant heart failure patients in the train set for both deceased and surviving groups

The results of the train set show that there were 321 patients in the survival group and 59 patients in the death group. The results indicate that the levels of TP, PCT, and pH in the deceased group were lower than those in the survival group, while WBC, DBIL, Mg^2+^, TT, and NT-ProBNP levels were higher in the survival group. Additionally, the usage rate of β-blockers in the survival group was significantly higher than in the deceased group. Detailed data can be found in Table 4 below. After assigning values to empirical variables, it was found that the percentage of patients in the death group with DBIL >8.6umol/L, TP < 65 g/L, K^+^ > 5.3 mmol/L, Mg^2+^ > 1.02 mmol/L, and pH < 7.35 was higher than in the survival group. The percentage of patients in the death group with K^+^ < 3.5 mmol/L, Mg^2+^ < 0.75 mmol/L, pH > 7.45, and using β-blockers was lower than in the survival group. Detailed results are shown in Table 5.。

**Table 4:** Comparison of original data between the survival and death groups in the train set.

Variables (Train)	Total (n = 380)	Survival (n = 321)	Death (n = 59)	χ^2^/t/Z	*P*
TP, Mean ± SD	60.44 ± 8.72	61.05 ± 8.50	57.13 ± 9.23	3.21	0.001
WBC, M (Q₁, Q₃)	11.38 (7.85, 15.90)	11.00 (7.70, 14.94)	12.50 (8.39, 18.50)	−2.19	0.029
Lymph, M (Q₁, Q₃)	4.79 (3.00, 6.75)	4.62 (3.00, 6.71)	5.30 (2.98, 7.15)	−0.86	0.389
PCT, M (Q₁, Q₃)	0.31 (0.22, 0.41)	0.31 (0.23, 0.42)	0.27 (0.17, 0.36)	−2.23	0.026
DBIL, M (Q₁, Q₃)	1.81 (0.30, 3.48)	1.60 (0.30, 3.10)	3.17 (0.84, 5.42)	−3.42	<0.001
K^+^, M (Q₁, Q₃)	4.18 (3.74, 4.59)	4.15 (3.74, 4.56)	4.32 (3.78, 4.95)	−1.76	0.079
Mg^2+^, M (Q₁, Q₃)	0.91 (0.84, 0.98)	0.90 (0.83, 0.97)	0.93 (0.88, 1.05)	−2.61	0.009
TT, M (Q₁, Q₃)	24.80 (22.20, 27.63)	24.60 (22.20, 27.20)	25.80 (22.25, 30.25)	−2.04	0.041
pH, M (Q₁, Q₃)	7.39 (7.31, 7.44)	7.39 (7.32, 7.45)	7.30 (7.21, 7.40)	−3.78	<0.001
NT-ProBNP, M (Q₁, Q₃)	2822.00 (1585.00, 4576.25)	2578.00 (1432.00, 3723.00)	6810.00 (4436.50, 8569.50)	−7.72	<0.001
Respiratory rate, M (Q₁, Q₃)	36.50 (30.00, 49.00)	37.00 (30.00, 49.00)	36.00 (26.50, 49.00)	−0.92	0.355
Race, n(%)				0.28	0.599
Other	237 (62.37)	202 (62.93)	35 (59.32)		
Han	143 (37.63)	119 (37.07)	24 (40.68)		
β-blocker, n(%)				5.27	0.022
No	268 (70.53)	219 (68.22)	49 (83.05)		
Yes	112 (29.47)	102 (31.78)	10 (16.95)		
ACEI, n(%)				3.38	0.066
No	301 (79.21)	249 (77.57)	52 (88.14)		
Yes	79 (20.79)	72 (22.43)	7 (11.86)		

**Note.** t: *t*-test, Z: Mann-Whitney test, χ^2^: Chi-square test; SD: standard deviation, M: Median, Q₁: 1st Quartile, Q₃: 3rd Quartile.

**Table 5:** Comparison of dummy variable assignment between the survival and death groups in the train set.

Variables	Total (n = 380)	Survival (n = 321)	Death (n = 59)	χ^2^	*P*
Race, n(%)				0.28	0.599
Other	237 (62.37)	202 (62.93)	35 (59.32)		
Han	143 (37.63)	119 (37.07)	24 (40.68)		
WBC, n(%)				5.72	0.057
[3.5, 9.5]	132 (34.74)	117 (36.45)	15 (25.42)		
<3.5	11 (2.89)	7 (2.18)	4 (6.78)		
>9.5	237 (62.37)	197 (61.37)	40 (67.80)		
Lymph, n(%)				0.24	0.886
[1.10, 3.20]	90 (23.68)	76 (23.68)	14 (23.73)		
<1.10	15 (3.95)	12 (3.74)	3 (5.08)		
>3.20	275 (72.37)	233 (72.59)	42 (71.19)		
PCT, n(%)				0.00	0.979
[0, 0.5]	341 (89.74)	288 (89.72)	53 (89.83)		
>0.5	39 (10.26)	33 (10.28)	6 (10.17)		
DBIL, n(%)				5.07	0.024
≤8.6	352 (92.63)	302 (94.08)	50 (84.75)		
>8.6	28 (7.37)	19 (5.92)	9 (15.25)		
TP, n(%)				–	0.021
[65, 85]	103 (27.11)	95 (29.60)	8 (13.56)		
<65	274 (72.11)	223 (69.47)	51 (86.44)		
>85	3 (0.79)	3 (0.93)	0 (0.00)		
K^+^, n(%)				9.48	0.009
[3.5, 5.3]	288 (75.79)	248 (77.26)	40 (67.80)		
<3.5	60 (15.79)	52 (16.20)	8 (13.56)		
>5.3	32 (8.42)	21 (6.54)	11 (18.64)		
Mg^2+^, n(%)				9.23	0.01
[0.75, 1.02]	278 (73.16)	236 (73.52)	42 (71.19)		
<0.75	38 (10.00)	37 (11.53)	1 (1.69)		
>1.02	64 (16.84)	48 (14.95)	16 (27.12)		
TT, n(%)				–	0.137
[16, 25]	203 (53.42)	176 (54.83)	27 (45.76)		
<16	2 (0.53)	1 (0.31)	1 (1.69)		
>25	175 (46.05)	144 (44.86)	31 (52.54)		
pH, n(%)				18.99	<0.001
[7.35, 7.45]	155 (40.79)	142 (44.24)	13 (22.03)		
<7.35	143 (37.63)	106 (33.02)	37 (62.71)		
>7.45	82 (21.58)	73 (22.74)	9 (15.25)		
NT-ProBNP, n(%)				–	0.424
[0, 125]	18 (4.74)	17 (5.30)	1 (1.69)		
(125, 1800)	6 (1.58)	6 (1.87)	0 (0.00)		
≥1800	356 (93.68)	298 (92.83)	58 (98.31)		
Respiratory rate, n(%)				4.92	0.086
1–12 months: [30, 40], 13–36 months: [25, 30]	151 (39.74)	131 (40.81)	20 (33.90)		
1–12 months: <30, 13–36 months: <25	50 (13.16)	37 (11.53)	13 (22.03)		
1–12 months: >40, 13–36 months: >30	179 (47.11)	153 (47.66)	26 (44.07)		
β-blocker, n(%)				5.27	0.022
No	268 (70.53)	219 (68.22)	49 (83.05)		
Yes	112 (29.47)	102 (31.78)	10 (16.95)		
ACEI, n(%)				3.38	0.066
No	301 (79.21)	249 (77.57)	52 (88.14)		
Yes	79 (20.79)	72 (22.43)	7 (11.86)		

**Note.** χ^2^: Chi-square test, -: Fisher exact.

### 3.4 Results of single-factor and multiple-factor logistic regression in the train set

Single-factor logistic regression results for infant heart failure patients who died during hospitalization showed that: WBC<3.5∗10^9/L (OR = 4.46), DBIL> 8.6umol/L (OR = 2.86), TP < 65 g/L (OR = 2.72), K^+^>5.3 mmol/L (OR = 3.25), pH < 7.35 (OR = 3.81), and Respiratory rate (1–12 months: <30 times/min and 13–36 months: <25 times/min, OR = 2.30) are potential risk factors, while the use of β-blockers (OR = 0.44) is a potential protective factor. The results of multiple-factor logistic regression showed that: TP < 65 g/L (OR = 2.34), pH < 7.35 (OR = 2.79), and Respiratory rate (1–12 months: <30 times/min and 13–36 months: <25 times/min, OR = 2.34) are independent risk factors. Detailed data can be found in Table 6.

**Table 6:** Presentation of single-factor and multiple-factor logistic regression results in the train set.

Variables	single-factor					multiple-factor				
	β	S.E	Z	*P*	OR (95%CI)	β	S.E	Z	*P*	OR (95%CI)
Race										
Other					1.00 (Reference)					
Han	0.15	0.29	0.53	0.599	1.16 (0.66–2.05)					
WBC										
[3.5, 9.5]					1.00 (Reference)					1.00 (Reference)
<3.5	1.49	0.68	2.18	0.029	4.46 (1.17–17.04)	1.2	0.74	1.61	0.107	3.32 (0.77–14.24)
>9.5	0.46	0.32	1.42	0.156	1.58 (0.84–2.99)	0.3	0.35	0.84	0.4	1.34 (0.68–2.68)
Lymph										
[1.10, 3.20]					1.00 (Reference)					
<1.10	0.31	0.71	0.43	0.666	1.36 (0.34–5.44)					
>3.20	−0.02	0.34	−0.06	0.948	0.98 (0.51–1.89)					
PCT										
[0, 0.5]					1.00 (Reference)					
>0.5	−0.01	0.47	−0.03	0.979	0.99 (0.39–2.47)					
DBIL										
≤8.6					1.00 (Reference)					1.00 (Reference)
>8.6	1.05	0.43	2.43	0.015	2.86 (1.23–6.68)	0.79	0.49	1.63	0.104	2.21 (0.85–5.73)
TP										
[65, 85]					1.00 (Reference)					1.00 (Reference)
<65	1	0.4	2.5	0.012	2.72 (1.24–5.94)	0.85	0.42	2.02	0.043	2.34 (1.03–5.33)
>85	−13.09	840.27	−0.02	0.988	0.00 (0.00 ∼ Inf)	−12.94	834.87	−0.02	0.988	0.00 (0.00 ∼ Inf)
K^+^										
[3.5, 5.3]					1.00 (Reference)					1.00 (Reference)
<3.5	−0.05	0.42	−0.11	0.91	0.95 (0.42–2.16)	−0.18	0.45	−0.39	0.695	0.84 (0.35–2.03)
>5.3	1.18	0.41	2.88	0.004	3.25 (1.46–7.24)	0.77	0.45	1.71	0.088	2.17 (0.89–5.26)
Mg^2+^										
[0.75, 1.02]					1.00 (Reference)					
<0.75	−1.88	1.03	−1.83	0.067	0.15 (0.02–1.14)					
>1.02	0.63	0.33	1.88	0.06	1.87 (0.97–3.60)					
TT										
[16, 25]					1.00 (Reference)					
<16	1.87	1.43	1.31	0.19	6.52 (0.40–107.33)					
>25	0.34	0.29	1.18	0.236	1.40 (0.80–2.46)					
pH										
[7.35, 7.45]					1.00 (Reference)					1.00 (Reference)
<7.35	1.34	0.35	3.86	<0.001	3.81 (1.93–7.53)	1.03	0.37	2.74	0.006	2.79 (1.34–5.82)
>7.45	0.3	0.46	0.65	0.515	1.35 (0.55–3.30)	0.3	0.47	0.63	0.526	1.35 (0.53–3.41)
NT-ProBNP										
[0, 125]					1.00 (Reference)					
(125, 1800)	−13.73	979.61	−0.01	0.989	0.00 (0.00 ∼ Inf)					
≥1800	1.2	1.04	1.15	0.249	3.31 (0.43–25.35)					
Respiratory rate										
1–12 months: [30, 40], 13–36 months: [25, 30]					1.00 (Reference)					1.00 (Reference)
1–12 months: <30, 13–36 months: <25	0.83	0.4	2.07	0.038	2.30 (1.05–5.06)	0.85	0.43	1.97	0.048	2.34 (1.01–5.44)
1–12 months: >40, 13–36 months: >30	0.11	0.32	0.33	0.738	1.11 (0.59–2.09)	−0.18	0.35	−0.52	0.604	0.83 (0.42–1.66)
β-blocker										
No					1.00 (Reference)					1.00 (Reference)
Yes	−0.83	0.37	−2.25	0.025	0.44 (0.21–0.90)	−0.52	0.4	−1.3	0.192	0.60 (0.27–1.30)
ACEI										
No					1.00 (Reference)					
Yes	−0.76	0.42	−1.8	0.071	0.47 (0.20–1.07)					

**Note.** OR: Odds Ratio, CI: Confidence Interval.

### 3.5 Evaluation and presentation of the predictive model for in-hospital mortality of infant heart failure patients

**Fig. 3:**
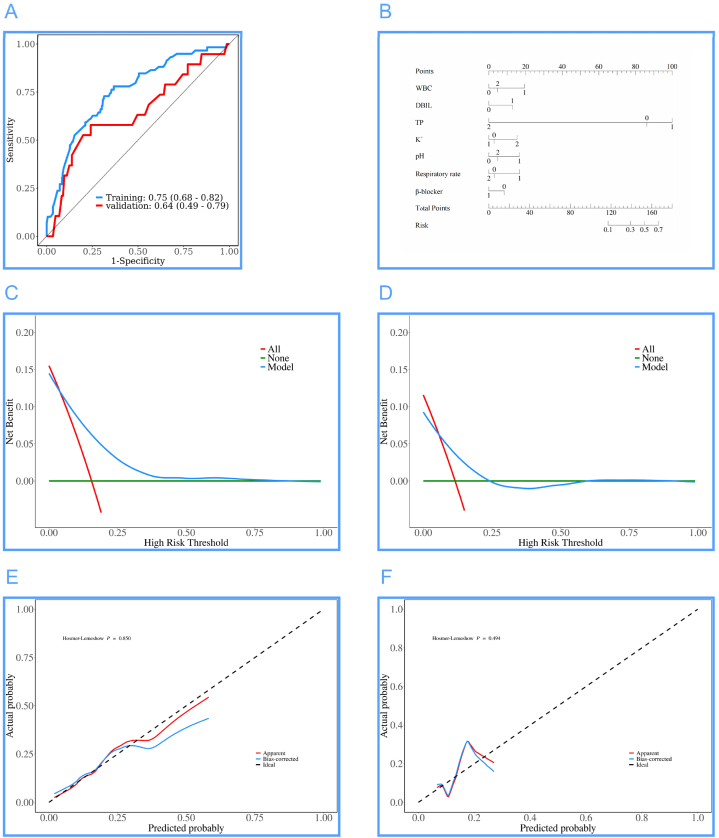
Results of the prediction model for in-hospital mortality in infants with heart failure. (A) ROC curves of the train and test sets, (B) Diagnostic nomogram of the mortality model, (C) DCA curves of the train set, (D) DCA curves of the test set, (E) Diagnostic calibration curves of the train set, (F) Diagnostic calibration curves of the test set.

Fig. 3A: The AUC of the train and test sets are 0.75 (0.68, 0.82) and 0.64 (0.49, 0.79) respectively, indicating good predictive performance of the model. Fig. 3B: The nomogram is based on the multiple-factor logistic regression analysis, where the scales corresponding to WBC, DBIL, TP, K^+^, pH, Respiratory rate, and β-blocker are marked on the respective segments, representing the range of variable values, showing that TP has the most significant impact on the prediction outcome. Fig. 3C and D: The DCA curves of the train and test sets both indicate a good application range of this model. Fig. 3E and F: The diagnostic calibration curves of the train and test sets further demonstrate the stable and accurate predictive performance of the model.

The evaluation results of the model for infant heart failure patients in the train and test sets are as follows: Likelihood ratio tests were employed to test the model, and both the train and test sets were significant, with P values of 5.53∗10^-5 and 0.0048 respectively. The C-index values for discrimination in the train and test sets were 0.721 and 0.728. Goodness-of-fit tests for calibration assessments showed P values of 0.9958 and 0.9998, both greater than 0.05, indicating good calibration. Detailed data can be found in Table 7.

**Table 7:** Model evaluation of infant heart failure patients in the train and test sets.

Evaluation direction		Train		Test	
	Evaluation content	Statistics	P	Statistics	P
Model verification	Likelihood ratio test	χ^2^: 33.262	5.53∗10^-5	χ^2^: 33.262	0.0048
Discrimination assessment	C-index	C-index: 0.721 (0.652–0.790)	9.84∗10^-10	C-index: 0.728 (0.652–0.790)	0.0001
Calibration assessment.	Goodness-of-fit test.	χ^2^: 5.4234	0.9958	χ^2^: 5.4234	0.9998

The AUCs of the train and test sets were 0.75 and 0.64, with accuracies of 0.69 and 0.68 respectively. The sensitivity and specificity of the train set were 0.69 and 0.73, while those of the test set were 0.70 and 0.58. The PPVs of the train and test sets were 0.93 and 0.93, and the NPVs were 0.30 and 0.20. These results indicate good predictive accuracy for in-hospital mortality of infant heart failure patients. Detailed data can be found in Table 8.

**Table 8:** Confusion matrix results of the train and test sets.

Data	AUC (95%CI)	Accuracy (95%CI)	Sensitivity (95%CI)	Specificity (95%CI)	PPV (95%CI)	NPV (95%CI)	Cut off
Train	0.75 (0.68–0.82)	0.69 (0.64–0.74)	0.69 (0.63–0.74)	0.73 (0.62–0.84)	0.93 (0.90–0.96)	0.30 (0.22–0.37)	0.148
Test	0.64 (0.49–0.79)	0.68 (0.61–0.75)	0.70 (0.62–0.77)	0.58 (0.36–0.80)	0.93 (0.88–0.98)	0.20 (0.09–0.31)	0.148

**Note.** PPV: Positive Predictive Value, NPV: Negative Predictive Value.

### 3.6 Scoring table and risk assessment for in-hospital mortality risk in infants with heart failure

The mortality risk scoring table is shown in Table 9. WBC >9.5∗10^9/L is assigned 1 point, DBIL >8.6 μmol/L is assigned 3 points, TP < 65 g/L is assigned 3 points, K^+^ > 5.3 mmol/L is assigned 3 points, pH < 7.35 is assigned 3 points, pH > 7.45 is assigned 1 point, Respiration rate (1–12 months: <30 times/min, 13–36 months: <25 times/min) is assigned 3 points, and the use of β-blocker is assigned −2 points.

**Table 9:** Risk scoring table for in-hospital mortality in infants with heart failure (used by train and test sets).

Characteristics	Categories	Points
WBC	>9.5∗10^9/L	1
DBIL	>8.6 μmol/L	3
TP	<65 g/L	3
K^+^	>5.3 mmol/L	3
pH	<7.35	3
pH	>7.45	1
Respiration rate, times/min	1–12 months: <30, 13–36 months: <25	3
β-blocker	Yes	−2

**Fig. 4:**
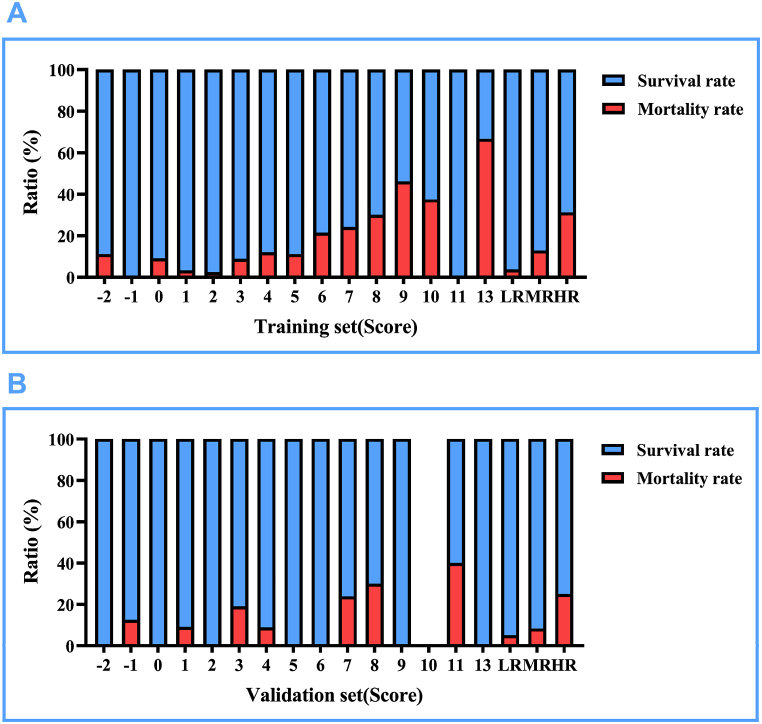
Presentation of mortality rates based on risk score values. LR: Low risk, MR: Medium risk, HR: High risk. (A) Display of survival and mortality rates for each score in the training set. (B) Display of survival and mortality rates for each score in the validation set.

After assigning scores to the train and test sets based on the scoring table, this study categorizes −2–2 points as low risk, 3–6 points as medium risk, and 7–13 points as high risk. An interesting observation is that in Fig. 4A and B, both the train and test sets show an increasing trend in mortality rates with higher scores, especially in the low-risk, medium-risk, and high-risk categories. Detailed data can be found in Fig. 4 and Table 10.

**Table 10:** Mortality and survival rates of infants with heart failure in the train and test sets at different scores.

Points	Train				Test			
	Life	Death	Mortality rate	Survival rate	Life	Death	Mortality rate	Survival rate
−2	8	1	11.11 %	88.89 %	2	0	0.00 %	100.00 %
−1	15	0	0.00 %	100.00 %	7	1	12.50 %	87.50 %
0	10	1	9.09 %	90.91 %	3	0	0.00 %	100.00 %
1	30	1	3.23 %	96.77 %	10	1	9.09 %	90.91 %
2	40	1	2.44 %	97.56 %	16	0	0.00 %	100.00 %
3	31	3	8.82 %	91.18 %	17	4	19.05 %	80.95 %
4	66	9	12.00 %	88.00 %	31	3	8.82 %	91.18 %
5	24	3	11.11 %	88.89 %	15	0	0.00 %	100.00 %
6	22	6	21.43 %	78.57 %	14	0	0.00 %	100.00 %
7	47	15	24.19 %	75.81 %	16	5	23.81 %	76.19 %
8	7	3	30.00 %	70.00 %	7	3	30.00 %	70.00 %
9	7	6	46.15 %	53.85 %	2	0	0.00 %	100.00 %
10	10	6	37.50 %	62.50 %	–	–	–	–
11	2	0	0.00 %	100.00 %	3	2	40.00 %	60.00 %
13	2	4	66.67 %	33.33 %	2	0	0.00 %	100.00 %
−2–2 points(Low risk)	103	4	3.74 %	96.26 %	38	2	5.00 %	95.00 %
3–6 points(Medium risk)	143	21	12.80 %	87.20 %	77	7	8.33 %	91.67 %
7–13 points(High risk)	75	34	31.19 %	68.81 %	30	10	25.00 %	75.00 %

The patients in the train and test sets are divided into low-risk, medium-risk, and high-risk groups based on their scores. Using the low-risk group as a reference, logistic regression results in the train set showed odds ratios of 3.78 and 11.67 for mortality risk in the medium-risk and high-risk groups, respectively; in the test set, the odds ratios for mortality risk in the medium-risk and high-risk groups were 1.73 and 6.33, respectively. Detailed data can be found in Table 11.

**Table 11:** Mortality and survival rates of infants with heart failure at different scores.

Variables	Train					Test				
	β	S.E	Z	*P*	OR (95%CI)	β	S.E	Z	*P*	OR (95%CI)
Sum										
−2–2 points(Low risk)					1.00 (Reference)					1.00 (Reference)
3–6 points(Medium risk)	1.33	0.56	2.37	0.018	3.78 (1.26–11.35)	0.55	0.83	0.66	0.508	1.73 (0.34–8.72)
7–13 points(High risk)	2.46	0.55	4.47	<0.001	11.67 (3.97–34.30)	1.85	0.81	2.27	0.023	6.33 (1.29–31.11)

注:OR: Odds Ratio, CI: Confidence Interval。.

## 4 Discussion

Infant heart failure, due to its complexity and multitude of factors, has resulted in high mortality rates during hospitalization. Because of this disease burden, it has also imposed heavy burdens on families, evolving into a significant public health issue. In this study, 14 variables were selected using Lasso regression from numerous patient information, including TP, WBC, Lymph, PCT, DBIL, K^+^, Mg^2+^, TT, pH, NT-ProBNP, Respiratory rate, Race, β-blocker, and ACEI/ARB/ARNI. Multiple-factor logistic regression results revealed that TP < 65 g/L (OR = 2.34), pH < 7.35 (OR = 2.79), and Respiratory rate (1–12 months: <30 times/min and 13–36 months: <25 times/min, OR = 2.34) were independent risk factors. A mortality risk scoring table was established for infants with heart failure during hospitalization, categorizing −2 to 2 points as low risk, 3 to 6 points as medium risk, and 7 to 13 points as high risk after assigning scores to the train and test sets. Interestingly, the train and test sets showed an increasing trend in mortality rates with higher scores, especially in the low-risk, medium-risk, and high-risk categories. The results partly elucidate the good predictive performance, accuracy, and value of this model, serving as an important reference for addressing the risk of mortality in infants with heart failure in China. It also provides a reliable theoretical basis for future clinical treatment of heart failure by healthcare professionals.

In this study, low serum total protein levels were found to be an independent risk factor for in-hospital mortality in infants with heart failure. Patients with heart failure often have poor nutritional status, which hinders protein synthesis and absorption in the body, leading to exacerbation of protein loss. This, coupled with activated inflammatory responses, may be one of the main causes of hypoalbuminemia [7]. A decrease in serum albumin levels can exacerbate fluid retention and tissue oedema, particularly pulmonary oedema, which may increase the risk of developing acute respiratory distress syndrome [8]. An increasing number of studies have confirmed that serum albumin levels have important predictive value in the prognosis of heart failure patients. After adjusting for baseline differences, lower serum albumin levels have been identified as an independent risk factor for increased all-cause mortality [9]. Bonilla-Paloma et al.'s study, which included 362 patients hospitalised for acute heart failure, found that after correcting for baseline differences, serum albumin levels emerged as a strong predictor for both in-hospital mortality and long-term mortality [10]. In this study, infants aged 1–36 months with TP < 65 g/L were found to have a 2.34 times higher risk of in-hospital mortality compared to patients with normal total protein levels, aligning with the results or phenomena discussed earlier. Therefore, patients with hypoalbuminemia also require careful attention from attending physicians.

It is evident that in this study, patients with a blood gas analysis result of pH < 7.35 experienced a 1.902 times increase in the risk of death, similar results have been observed in other studies, indicating that patients with heart failure may develop acidosis, thereby increasing the risk of mortality [11]. Our analysis suggests that this may be due to the decreasing left ventricular ejection ability in heart failure patients, leading to reduced cardiac output. The decrease in left ventricular output results in inadequate blood and oxygen supply to the organs and tissues, triggering anaerobic metabolism and lactic acid accumulation, making heart failure patients prone to metabolic acidosis. Acidosis in patients is often accompanied by electrolyte imbalances, therefore, in clinical practice, when children experience metabolic acidosis, it is important to correct it promptly and properly while considering the status of electrolyte disturbances.

The condition of infant heart failure may be associated with changes in respiratory rate and mortality rates. In cases of infant heart failure, a decrease in respiratory rate may indicate increased disease severity, worsening cardiac function, or inadequate oxygen supply. Compared to adults, changes in respiratory rate may be more sensitive in infants with heart failure as their physiological functions are not fully developed, which could have a more significant impact on cardiac function. Overall, a decrease in respiratory rate in infants with heart failure may be associated with an increased risk of mortality [12]. This study also confirmed this, showing that infants with heart failure who had a decreased respiratory rate had a 2.769 times increased risk of mortality. Therefore, timely monitoring and addressing abnormal respiratory rates are crucial for improving survival rates and prognosis in infants with heart failure.

In addition to the aforementioned risk factors serving as independent risk factors for in-hospital mortality in children, there are other factors that may have an impact on the mortality of infants with heart failure during hospitalization. The established mortality scoring table indicates that high levels of WBC, DBIL, and K+ are risk factors for death, while taking β-blockers is a protective factor. For patients with elevated white blood cell counts, it is often associated with factors such as viral infections, weakened immune function, and the use of diuretics, among others. The cachexia state resulting from these factors can increase the mortality rate of heart failure [13]. Patients with heart failure often experience impaired left ventricular filling function or reduced left ventricular ejection fraction, leading to inadequate multi-organ blood perfusion or congestion, resulting in organ dysfunction and, particularly, liver and kidney damage [14]. The results of this study also show that heart failure patients who died had high levels of serum direct bilirubin. Research has also indicated that hyperkalaemia has a significant impact on the adverse prognosis of heart failure patients [15]. ACEIs/ARBs/ARNIs and β-blockers are currently the cornerstone medications for heart failure patients, antagonising the RAAS system and the sympathetic nervous system, playing important roles in reversing ventricular remodelling [16]. These medications have shown good effects in improving the prognosis and reducing the occurrence of long-term adverse outcomes in paediatric heart failure patients. The results of this study further elucidate this point, demonstrating that the use of ACEIs/ARBs/ARNIs and β-blockers is beneficial in combating mortality in hospitalised children [17].

Overall, the purpose of this retrospective study was multifaceted. Firstly, it aimed to identify the influencing factors for in-hospital mortality in infants with heart failure, whether they are protective factors or risk factors. On the other hand, we aimed to establish a predictive model for in-hospital mortality in children, and the results showed that the model had good accuracy and validity. Additionally, the scoring table established based on the predictive model in this study provided a good evaluation and predictive effect for in-hospital mortality, which can serve as valuable guidance and reference for future clinical treatment.

## 5 Strengths and limitations

Although this study conducted a significant amount of work within a limited scope, there are still several limitations. Despite retrospectively collecting data from all paediatric heart failure patients hospitalised from 2012 to 2023, the sample size of 544 cases is still relatively small. Secondly, from an evidence-based medicine perspective, retrospective studies are considered to have lower levels of evidence compared to prospective studies. However, this will be one of our main focuses for future research. Thirdly, while this hospital is the largest regional hospital with the highest number of inpatients, this study is limited to being conducted at a single centre. The results obtained may not fully represent the hospitalization situations of paediatric heart failure patients in China, as the findings were only internally validated at this centre and not externally validated in other hospitals or regions. These limitations serve as the driving force for our future research efforts, as we aim to conduct prospective multicentre studies to provide assistance to a wider range of patients and healthcare professionals.

## 6 Conclusion

In conclusion, the independent risk factors leading to in-hospital mortality in children are low TP, pH, and respiratory rate. When combined with our scoring table, it also shows good predictive efficacy for the mortality risk in paediatric heart failure patients during hospitalization.

## CRediT authorship contribution statement

**Meng Wei:** Writing – review & editing, Writing – original draft, Visualization, Supervision, Software, Project administration, Investigation, Data curation, Conceptualization. **Shuai Shang:** Formal analysis. **Huasheng Lv:** Validation, Investigation. **Xiaoyan Liang:** Validation, Software. **Yanmei Lu:** Supervision, Methodology, Funding acquisition. **Baopeng Tang:** Resources, Funding acquisition, Conceptualization.

## Data availability statement

Data will be made available on request.

## Declaration of competing interest

The authors declare no conflict of interest.
